# Factors associated with tungiasis among primary school children: a cross-sectional study in a rural district in Rwanda

**DOI:** 10.1186/s12889-019-7481-y

**Published:** 2019-08-29

**Authors:** Jerome Nsanzimana, Simon Karanja, Moses Kayongo, Naphtal Nyirimanzi, Hyacinthe Umuhoza, Anthère Murangwa, Raymond Muganga, Aimable Musafili

**Affiliations:** 1 Butare University Teaching Hospital, Huye, Rwanda; 2 Medical Epidemiology, School of Public Health, Jomo Kenyatta University of Agriculture and Technology, Nairobi, Kenya; 3 School of Public Health, Jomo Kenyatta University of Agriculture and Technology, Kigali, Rwanda; 4 Department of Infection Prevention and Control, Kigeme District Hospital, Nyamagabe, Rwanda; 5 Rwanda Military Hospital, Kigali, Rwanda; 6 College of Medicine and Health Sciences, University of Rwanda, Kigali, Rwanda

**Keywords:** Tungiasis, Primary school children, Rural area, Rwanda

## Abstract

**Background:**

Tungiasis is a relatively frequent ectoparasitosis in low-income settings, yet its morbidity and social impact are still not well understood due to the scarcity of information. In Rwanda, data on the magnitude and conditions leading to the tungiasis is rare. This study sought to determine the prevalence and factors associated with tungiasis among primary school children in Rwandan setting.

**Method:**

A descriptive cross-sectional study utilising systematic random sampling method was adopted to select 384 children from three primary schools. From July to October 2018, data were collected on socio-demographic characteristics of children, parents, and households. Logistic regression was applied to analyse socio-demographic factors associated with tungiasis with a level of significance set at *P*-value< 0.05.

**Results:**

Prevalence of tungiasis among three primary schools was 23%. Factors associated with tungiasis included walking barefoot (AOR: 78.41; 95% CI: 17.91–343.10), irregular wearing of shoes (AOR: 24.73; 95% CI: 6.27–97.41), having dirty feet (AOR: 12.69; 95% CI: 4.93–32.64), wearing dirty clothes (AOR: 12.69; 95% CI: 4.18–38.50), and living in a house with earthen plastered floor (AOR: 28.79; 95% CI: 7.11–116.57). Children infected with tungiasis attended class less frequently (AOR: 19.16, 95%CI: 7.20–50.97) and scored lower (AOR: 110.85, 95%CI: 43.08–285.20) than those non-infected. The low school attendance and poor performance could be partly explained by difficulty of walking, lack of concentration during school activities, and isolation or discrimination from classmates.

**Conclusion:**

Tungiasis was a public health challenge among school going children in a rural Rwandan setting. This study revealed that children affected with tungiasis had poor hygiene, inadequate housing environments and consequently poor school attendance and performance. Improving socio-economic conditions of households with special emphasis on hygiene of family members and housing conditions, would contribute to preventing tungiasis.

**Electronic supplementary material:**

The online version of this article (10.1186/s12889-019-7481-y) contains supplementary material, which is available to authorized users.

## Background

Tungiasis is an ectoparasitosis, which is caused by infestation with *Tunga penetrans*, also known as a jigger flea or sand flea. It is usually considered as an entomologic nuisance, yet it does not receive much attention from healthcare professionals. Tungiasis, as a zoonotic disease, increases the parasite burden in human, resulting in considerable debilitating morbidity in endemic areas [1, 2].

Tungiasis develops through a life cycle, which consists of an on-host and off-host phase. The on-host phase starts by penetration of a non-fertilised female sand flea into the epidermis, where it stays permanently and feeds on the blood of its host [3, 4]. After penetrating the skin, the female sand flea becomes hypertrophic, following the intestine growth and maturation of eggs in the abdomen [3, 5]. The female sand flea is fertilised by a male through a tiny opening of the last abdominal segment, which maintains the parasite in contact with the environment [3]. This opening is also used by the parasite to breathe, defecate, and expel eggs to the outside after copulation with the male sand flea and may serve as an entry point for microbes [6].

After expelling eggs, the female sand flea dies and is discarded from the damaged skin tissue during the healing process [3]. The on-host phase develops in humans or animal reservoirs, including various domestic and wild animal species, in which embedded sand fleas can develop. In some settings, dogs, cats, pigs, cattle, sheep, goats, monkeys, and sylvatic rodents have been found as reservoirs of *Tunga penetrans* [7–9].

The off-host phase starts by the expulsion of eggs to the environment. These eggs may be transported to the cracks and holes of the house when cleaning the floor. In a favourable environment, eggs undergo different developmental stages, including larval, pupal, and adult stage. The development of these stages may take several months [4, 10].

People acquire tungiasis when they walk barefoot or sleep on the ground, which harbour sand fleas. Almost all embedded sand fleas affect feet even though ectopic penetration sites are also reported [11, 12]. In rural Africa, classrooms containing crevices and holes are known to be ideal places for proliferation and transmission of *Tunga penetrans* [13]. Several studies in low and middle-income countries have shown the highest prevalence of tungiasis in poor communities in South America and Africa [14, 15]. In these communities, risk factors for tungiasis include poor body hygiene, living in a house with unhealthy conditions, sharing house with domestic animals, and the lack of education on disease prevention [1, 16–18]. Tungiasis is also prevalent among children, especially boys, and elderly people [13, 19–21].

Tungiasis is characterised by acute clinical manifestations, resulting from the inflammatory response to the burrowed female sand fleas and bacterial superinfection, which aggravates lesions [3, 6]. These manifestations include white nodules with black centres located in the affected body areas such as feet, toes, fingers, and interdigital spaces [11, 12]. Inflammatory signs such as oedema and skin redness around lesions of tungiasis may also be identified. Patients experience itching spots or intense pain, which can alter the walking. Other manifestations include suppurative ulcers and punctiform cavities, which occur after *Tunga penetrans* removal by patients or caretakers using needles or thorns [3, 6, 22–25]. The removal of embedded sand flea by inappropriate instruments may also lead to severe complications such as gangrene, sepsis, and infection by Clostridium tetani in non-vaccinated individuals [23].

In addition to acute manifestations, patients affected by tungiasis may develop sequels, including chronic pain, loss of toes and nails, deformation of toes and nails, formation of fissures, and mutilation of feet, which leads to walking difficulties [26, 27]. Acute and chronic manifestations, and social stigma associated with these manifestations make it hard for school children to reach school and attend class, concentrate in class, or remain in the school. These difficulties may lead to poor school performance and failure to proceed to next classes [20, 26, 28, 29].

In Rwanda, neglected diseases, including tungiasis, have drawn special attention of stakeholders in recent years. Programs aiming at preventing and treating tungiasis at community level have been strengthened. These programs include public education and sensitisation on prevention of tungiasis, removal of *Tunga penetrans*, and cleaning the wound left by tungiasis using appropriate disinfectant [30, 31]. However, little is still unknown regarding prevalence and underlying factors of this infestation, especially among children, who constitute a vulnerable group to the infestation. The aim of this study was to assess the prevalence and socio-demographic factors associated with tungiasis among children of school age in a rural area.

## Methods

### Design

This was a cross-sectional study, where primary school children living in a rural area were assessed for socio-demographic characteristics (exposures) and tungiasis infestation (outcome) during the study period.

### Setting

The study was conducted in a Southern rural district in Rwanda, which was populated by 322,506 inhabitants and counted one of the highest poverty rates in the country [32]. Most inhabitants were farmers living on agriculture and livestock farming. Animals raised included cows, pigs, goats, hens, and pets such as dogs and cats. Specific interventions to eradicate tungiasis were operational in this district. Children affected by this ectoparasitosis underwent specific treatment, consisting of *Tunga penetrans* removal and wound care using disinfectants. On average, children had to walk a distance of two kilometres to reach primary schools from their homes.

Children included in the study were selected from three primary schools located in remote rural area, where the likelihood to find cases of children infected with tungiasis could be high. These schools were purposively selected following a non-random process and were not distant from each other to limit our expenses. In these schools, 71% of classes had walls built in cooked bricks or block cement whereas 29% of remaining classes had walls made of block mud. All three schools had cemented floors and walls. Fissures were noticed in the floors and walls of some classes, especially those having walls built in block mud. In 69% of cases, the roofs of classes were covered by iron sheets whereas 31% of classes were covered by traditional roofing tiles. Each class had a surface area estimated at 56m^2^ and could accommodate 50 pupils on average.

### Population and sample size calculation

The total number of pupils was 1603 in primary school A,1047 in primary school B, and 702 in primary school C. To calculate the adequate sample size, the following formula indicated for prevalence study was applied: $n=\frac{Z^2\mathrm{P}\left(1-\mathrm{P}\right)}{e^2}$ [33], where n was a minimal required sample size, z a standard normal deviation for a 95% confidence interval, p an estimated prevalence of tungiasis, and e the degree of precision. The prevalence was estimated by random at 50% since no previous study on tungiasis was done in Rwanda. Thus, for z = 1.96, *p* = 0.5, and e = 0.05, n was estimated at 384 children. These children were recruited from the three schools with equal number of 128 pupils per school. We included children aged 5 years or above, who were registered and attended classes at three primary schools involved in the study. Their families should live in a rural district involved in this research. All children aged below 5 years or whose families lived in another district were excluded from the study.

### Sampling technique

When selecting the study population, the skip interval approach, which is a systematic sampling, was applied. This type of sampling is a probability-based sampling method by which a study population is selected from larger population, using a random starting point and a fixed periodic interval. This interval is obtained by dividing the larger population size by the calculated sample size.

The skip interval applied in this study was equal to the total number of pupils at each school divided by 128. Starting from primary school one to primary school three, all registered pupils were identified using an alphabetic order. Then, they were assigned ordinal numbers corresponding to their alphabetic order. These numbers were put and mixed into a basket before randomly picking up the first number to start with. After selecting this number, corresponding to the first participant, the remaining participants were selected after skipping a constant interval specific to each school. For example, at a primary school A, the applied skip interval was 12 with a starting point number 51. With this skip interval, the next selected pupil was the 64th child followed by the 77th, and so on. The same approach was applied to other schools until the end of selection process.

### Data collection

After obtaining parental consent for participation in the research, children, who were included in the study, were clinically assessed by inspection to ascertain whether they had acute or chronic lesions suggesting tungiasis infestation, as described above. This assessment was performed at their homes. A case definition of tungiasis referred to the clinical findings associated with tungiasis, as reported in other studies [3, 27]. At least one embedded sand flea was also considered when defining a case of tungiasis.

Data collection also focused on demographic characteristics of children, which included their age and gender. Mothers or fathers were interviewed about maternal education, fathers’ occupation, and households’ conditions. Characteristics of the environmental housing, where children lived, were recorded through observations made during home visits. Information related to the situation of children at school was obtained from their teachers. Data collection was done using a structured questionnaire (see Additional file 1), which was tested before the actual data collection for possible readjustments. Information collected through this testing was not included in the study.

### Outcome and independent variables

The outcome of the study was tungiasis, which was recorded as a dichotomous variable based on having infestation or not.

Independent variables included socio-demographic characteristics of children, parents, households’ conditions, environmental housing, and situation at school. Gender was defined as male or female. Age was categorised into three groups as follows: 5–10 years, 11–15 years, and > 15 years. In Rwanda, regular pupils finish the primary school at the age of 12 years. However, due to multiple school interruptions related to chronic illnesses or extreme poverty, some pupils may delay completing their studies. Maternal education was defined as the highest level of education completed and was split into three categories, including no schooling, primary, secondary and above. In a Rwandan educational system, secondary school lasts 6 years.

Maternal education was considered as the reference in helping children based on a critical role that mothers are playing in child education. Mothers are considered as the first teachers of their children, which is the base of better future life of the children [34, 35]. Thus, educated mothers may help family, especially children by providing basic education in diseases prevention, which is crucial for child health and survival.

Occupation of the father was categorised in four groups, including farmer, trader, monthly salaried, occasional occupation, and not specified activity. Later these groups were condensed into two categories: farmer and other occupation. In low-income countries, fathers, also considered as breadwinners, play a vital role in providing financial support to their families, which contributes to their welfare. Thus, father’s occupation was taken as reference since it is considered as an important economical resource of the family, which helps cater for family needs [36].

Body’s hygiene was assessed by looking whether feet and clothes were dirty or clean. Dirty feet or clothes were those having dust or not washed. Wearing shoes regularly was considered as putting on always shoes when at home or elsewhere. Wearing shoes irregularly was considered as putting on shoes only when going to school or special circumstances such as going to the church or other social events while walking barefoot was considered as never wearing shoes.

Households’ conditions were categorised based on possession of domestic animals (cows, goats, pigs, rabbits, hens, and cats), and sharing houses with these animals. Proxies used to describe environmental housing included types of materials used to build floors (mud or earthen, cemented) and walls (mud or earthen, cemented). Situation at school was assessed through school attendance. Regular attendance was considered as never or rarely missing the class. Irregular attendance was defined as missing one or more days per week repeatedly within a period of last 6 months. This information was collected from the attendance register of each class and interviews with teachers. The performance of children in classes was expressed by average scores obtained from the first two trimesters (< 50%, ≥50%), taking the ongoing school year as a reference. These scores were found on the school transcripts. The scores for the third trimester were not yet available during the data collection. Possible reasons for school absenteeism in children affected by tungiasis were also investigated.

### Statistical analysis

Initially, descriptive statistics were performed using frequency tables and cross tabulations. Chi-square test was applied to assess differences between categories of different variables. The association between independent variables and outcome was analysed using logistic regression. Bivariate logistic analysis (crude analysis) was used to determine the strength of association between independent variables and outcome at the first instance. Variables with significant association in the bivariate analysis were included in the multivariate logistic regression (adjusted analysis). The independent variables were grouped into two categories-factors potentially leading to tungiasis such as dirty feet or clothes and consequences of tungiasis such as poor performance at school. Two models were formulated based on these two types of variables. Results were presented as adjusted odds ratio (AOR) with a 95% confidence interval (CI). All analyses were performed using IBM SPSS Statistics 20.0.

## Results

**Fig. 1:**
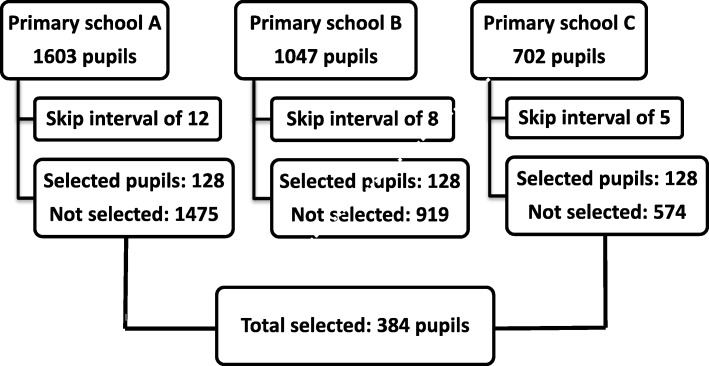
Flowchart of the study population

In total, 384 school children were included in the study, as indicated in the Fig. 1. Out of these, 87 (23%) were infected with tungiasis. Parents or caretakers and teachers at primary schools, successfully completed interviews conducted by the principal investigator with a response rate of 100%. No missing information was recorded. Most children were girls (51%). Age of participants was ranged between 6 and 18 years with the mean of 11 years and standard deviation of 2.6 years.

The characteristics of children included in the study were reported in the Table 1. The highest proportions of children infected with tungiasis were found among boys, and children whose mothers had only completed a primary school, those whose fathers were farmers, those who had dirty feet or wore dirty clothes or walked barefoot, those whose families shared houses with domestic animals, and those who lived in houses with earthen floors. The proportions of children infected with tungiasis were similar between various age groups of children.

**Table 1:** Socio-demographic characteristics of participants from a rural district, Rwanda, July to October 2018

Socio-demographic characteristics	Infected with tungiasis		Non-infected with tungiasis		Chi-square	*P*-value
	*n* = 87	%	*n* = 297	%		
Sex						
Male	52	60	138	46	5.62	0.02
Female	35	40	159	54		
Age						
5–10 years	36	41	156	53	3.51	0.17
11–15 years	42	48	123	41		
> 15 years	9	10	18	6		
Maternal education						
Secondary and above	3	3	30	10	6.70	0.04
Primary	53	61	194	65		
No schooling	31	36	73	25		
Father occupation						
Farmer	67	77	236	79	0.24	0.66
Other occupation	20	23	61	21		
Body’s hygiene						
Dirty feet	70	81	98	33	62.77	0.01
Clean feet	17	20	199	67		
Clothes’ hygiene						
Dirty clothes	65	75	92	31	53.25	0.01
Clean clothes	22	25	205	69		
Wearing shoes						
Barefoot	51	59	27	9	109.73	0.01
Irregularly	31	35	147	50		
Regularly	5	6	123	42		
Domestic animals						
Sharing house with domestic animals	71	82	187	63	10.61	0.01
Not sharing house with domestic animals	16	18	110	37		
Type of domestic animals						
Goats	26	30	89	30	18.35	0.01
Pigs	21	24	34	11		
Hens						
Cows	5	6	10	3		
Rabbit	5	6	13	4		
Cats	2	2	14	5		
No domestic animals at home	16	18	110	37		
Plastering of the house floor						
Earthen floor	81	93	206	69	20.09	0.01
Cemented floor	6	7	91	31		
School attendance						
Regular school attendance or missed days per week	68	78	258	87	4.00	0.04
Missed class more than two days per week	19	22	39	13		
School performance						
Scored ≥50%	25	29	275	93	160.55	0.01
Scored < 50%	62	71	22	7		

Irregular school attendance and poor performance were prevalent among children infected with tungiasis. Reasons related to the school absenteeism and poor performance included difficulty of walking due to pain, itching disturbing the concentration in class, and isolation or stigma from classmates. These reasons were reported by 19% of children and caretakers. Unknown or other social reasons for school absenteeism were identified among 47% of children with tungiasis.

Crude and adjusted odds ratios for factors associated with tungiasis were reported in Tables 2 and 3. Table 2 focuses on potential risk factors of tungiasis while Table 3 illustrates factors considered as consequences of being infected by tungiasis among school children. In Table 2, some factors remained associated with tungiasis in both crude and adjusted analyses. These included dirty feet, dirty clothes, walking barefoot, irregular wearing of shoes, and living in a house with earthen floor. Other factors, which were associated with tungiasis in crude analysis, lost their significance levels after adjustments. These factors included the sex of child, maternal education limited to primary school, sharing a house with domestic animals, and possession of domestic animals such as pigs, cows, and hens. Neither child age nor father occupation was associated with tungiasis in crude analysis. Table 3 shows that children infected with tungiasis were more prone to the school absenteeism and scored lower in classes than their classmates.

**Table 2:** Crude and adjusted odds ratios for potential risk factors of tungiasis among school children from a rural district, Rwanda, July to October 2018

Variables	Crude analysis		Adjusted analysis	
	OR	95% CI	OR	95% CI
Sex				
Male	1.80	1.10–2.92	1.75	0.76–4.00
Female	Reference			
Age				
5–10 years	0.46	0.08–2.61		
11–15 years	0.72	0.13–4.02		
> 15 years	Reference			
Maternal education				
Secondary and above	Reference			
Primary	2.73	0.80–9.30	1.27	0.29–5.55
No schooling	4.24	1.20–14.95	4.23	0.67–26.41
Father occupation				
Farmer	1.15	0.65–2.05		
Other occupation	Reference			
Body’s hygiene				
Dirty feet	8.23	4.60–14.74	12.69	4.93–32.64
Clean feet	Reference			
Clothes’ hygiene				
Dirty clothes	6.58	3.82–11.32	11.86	4.18–38.50
Clean clothes	Reference			
Wearing shoes				
Barefoot	46.46	16.94–127.39	78.41	17.91–343.10
Irregularly	8.95	4.88–16.42	24.73	6.27–97.41
Regularly	Reference			
Domestic animals				
Sharing house with domestic animals	2.61	1.44–4.71	0.52	0.21–1.25
Not sharing house with domestic animals	Reference			
Type of domestic animals				
Goats	2.01	1.01–3.97	1.40	0.41–4.71
Pigs	4.24	1.99–9.03	0.59	0.09–3.91
Hens	3.05	1.29–7.21	0.51	0.07–3.62
Cows	3.43	1.04–11.35	0.50	0.03–7.78
Rabbit	2.64	0.83–8.41	3.10	0.62–15.56
Cats	4.32	0.20–4.72	1.75	0.26–11.74
No domestic animals at home	Reference			
Plastering of the house floor				
Earthen floor	5.96	2.52–14.16	28.79	7.11–116.57
Cemented floor	Reference

**Table 3:** Crude and adjusted odds ratios for factors considered as consequences resulting from tungiasis infestation among school children from a rural district, Rwanda, July to October 2018

Variables	Crude analysis		Adjusted analysis	
	OR	95% CI	OR	95% CI
School attendance				
Regular school attendance or missed class less than two days per week	Reference			
Missed class more than two days per week	1.85	1.00–3.40	19.16	7.20–50.97
School performance				
Scored ≥50%	Reference			
Scored < 50%	31.00	16.42–58.54	110.85	43.08–285.20

## Discussion

The objective of this study was to determine factors associated with tungiasis among primary school children living in a rural district in Rwanda over a period of 3 months. The prevalence of tungiasis in this population was relatively high. The study showed that tungiasis was associated with dirty feet, wearing dirty clothes, walking barefoot, irregular wearing of shoes, and living in a house with earthen floor. Increased risks of school absenteeism and poor performance were also found among children infected with tungiasis.

Tungiasis is neglected tropical disease mostly found in the low-resource communities of Africa, south America and Caribbean [37, 38]. Overall, figures of prevalence in these settings vary depending on the level of endemicity of tungiasis and the scope of study.

In our study, prevalence of tungiasis among primary school children living in a rural district of Rwanda was the same (23%) as the one found in a population-based study conducted in the Eastern region of Uganda [16]. In contrast, this figure was nearly a half of that found in a study carried out in rural communities in Nigeria, where the prevalence of tungiasis was 43% [38]. Similarly, a slightly higher proportion of children infected with tungiasis (48%) was shown by a study conducted among primary school children younger than 15 years of age in an endemic area of the Eastern region in Kenya [39]. However, in this country, other studies conducted on tungiasis among school children aged between 5 and 14 years showed a prevalence, which fluctuated between 19 and 44% [40, 41]. Further, in our study, the highest prevalence of tungiasis was found among boys. A similar finding was obtained by other authors in low- and middle-income countries [20, 29, 42]. This could be related to the types of sports or games played by boys such as soccer, which may expose them to the contact with the soil and sand flea.

In this study, risk factors for *Tunga penetrans* were similar to those reported in previous studies. These factors included walking barefoot, irregular wearing of shoes, wearing only sandals, and low socioeconomic status [22, 43]. Poverty and ignorance could explain the lack of interest to use footwear. Other studies showed that having dirty feet or wearing dirty clothes, living in a house with earthen plastered floor and cracks in walls increased risks for tungiasis infestation [16, 39]. People living in these conditions could be exposed to the tungiasis by walking or sleeping on the houses floors [44]. Thus, better housing conditions could contribute to the prevention of risks for tungiasis infestation [22, 45].

Unlike to our findings, studies conducted elsewhere have demonstrated that low educational levels of mothers and sharing houses with domestic animals increased risks of contracting tungiasis. In line with this, an increased risk of developing tungiasis among primary school children in Ethiopia was found among children whose mothers were uneducated [46]. This might be related to the poverty and ignorance of these mothers, who could be short of financial means necessary to cater for basic needs of their children such as purchasing shoes and clothes for them.

Previous studies have also shown that domestic animals, including pigs, dogs and cats were reservoirs of *Tunga penetrans* and important sources of tungiasis infestation to the human [17, 47]. This was confirmed by a study conducted in Uganda, which showed that tungiasis mainly affected pigs and dogs [47, 48]. Likewise, studies done in Tanzania and Northeast of Brazil reported that living in the same houses with domestic animals constituted a major source of tungiasis for family members [17, 20]. Other reports indicate that in tropical settings, especially in rural areas, human beings and domestic animals are closely together, yet some people are not aware of tungiasis infestation among domestic animals, which may lead to higher risks of contracting this zoonotic disease [47, 49].

In our study, low educational level of mothers and sharing houses with domestic animals were associated with tungiasis in crude analysis but this association was diluted after adjustments. This could be partly explained by a relatively low sample size compared to the high number of variables put into the same model when doing adjusted analysis.

This study showed that tungiasis infestation was a risk, which led to missing classes, low participation in school activities, and poor performance at primary schools. The same risk was reported in other countries, where school going children were infected with tungiasis [29, 50].

This study on prevalence and factors associated with tungiasis among primary school children living in a rural area was the first of its kind conducted in a Rwandan setting. To minimize any misclassification, criteria required for a case definition of tungiasis were applied throughout the study. This helped to prevent any underestimate or overestimate of the number of tungiasis cases.

The screening of tungiasis was based on clinical skin lesions observed through inspection without using a magnifying lens. However, it is recommendable to use such a lens for better visualising these lesions. In this study, potential recall bias was prevented through interviews with parents or caretakers during home visits, where the investigator could explore home environment by direct observations.

The sample size was calculated using a standard formula applied for prevalence or cross-sectional studies. However, we believe that a bigger sample would have been necessary to counteract effects of adjustments, which resulted from the loss of significance of some variables and wide confidence intervals. In addition, when selecting participants, equal number was picked from each school regardless of the size of its population. This procedure might have introduced a selection bias in this study though the skip interval approach may have contributed to minimising this bias.

The population involved in this study was recruited from only three primary schools located in a rural area. Thus, our findings could not reflect the burden of tungiasis in the country as a whole. However, these findings may provide further insights regarding the magnitude and risks of tungiasis among children living in similar settings.

## Conclusion

Tungiasis infestation was an important health problem among primary school children in rural district of Rwanda. The prevalence of tungiasis was relatively high in this population. The presence of this neglected disease was associated with factors, including dirty feet, dirty wearing dirty clothes, walking barefoot, irregular wearing of shoes, and living in a house with earthen plastered floor. Tungiasis infestation was also associated with higher risks of absenteeism and poor performance at school. Thus, educating responsible people and families about body and clothes hygiene, regular use of footwear, and improvement of housing conditions such as plastering the house floor may be key strategies for preventing tungiasis and its burden.

## Additional file

Additional file 1: Research questionnaire (DOCX 49 kb)

## Data Availability

The datasets used and/or analysed during the current study are available from the corresponding author on reasonable request.

## Abbreviations

AOR: Adjusted odd ratio
CI: Confidence Interval
IBM: International Business Machines Corporation
OR: odd ratio
SPSS 20: Statistical Package for the Social Sciences 20

## References

[1] Mutebi F, Krücken J, Feldmeier H, Waiswa C. Animal Reservoirs of Zoonotic Tungiasis in Endemic Rural Villages of Uganda. PLOS Neglected Trop Dis. 2015:1–23. 10.1371/journal.pntd0004126. 10.1371/journal.pntd.0004126 PMC4608570 26473360

[2] Feldmeier H, Keysers A (2013). Tungiasis - a Janus-faced parasitic skin disease. Travel Med Infect Dis.

[3] Eisele M, Heukelbach J, Van Marck E, Mehlhorn H, Meckes O, Franck S, et al. Investigations on the biology, epidemiology, pathology and control of *Tunga penetrans* in Brazil: I. Natural history of tungiasis in man. Parasitol Res [Internet]. 2003;90:87–99. Available at: http://link.springer.com/10.1007/s00436-003-0950-2 10.1007/s00436-002-0817-y 12756541

[4] Nagy N., Abari E., D’Haese J., Calheiros C., Heukelbach J., Mencke N., Feldmeier H., Mehlhorn H. (2007). Investigations on the life cycle and morphology of Tunga penetrans in Brazil. Parasitology Research.

[5] Feldmeier H, Sentongo E, Krantz I (2013). Tungiasis (sand flea disease): a parasitic disease with particular challenges for public health. Eur J Clin Microbiol Infect Dis.

[6] Feldmeier H (2002). Bacterial superinfection in human tungiasis. Trop Med Int Heal..

[7] Pilger D, Schwalfenberg S, Heukelbach J, Witt L, Mehlhorn H, Mencke N (2008). Investigations on the biology, epidemiology, pathology, and control of Tunga penetrans in Brazil: VII. The importance of animal reservoirs for human infestation. Parasitol Res.

[8] Heukelbach J, Costa AML, Wilcke T, Mencke N, Feldmeier H (2004). The animal reservoir of Tunga penetrans in severely affected communities of north-East Brazil. Med Vet Entomol.

[9] Pampiglione S, Fioravanti ML, Gustinelli A, Onore G, Mantovani B, Luchetti A (2009). Sand flea (Tunga spp.) infections in humans and domestic animals: state of the art. Med Vet Entomol.

[10] Linardi PM, Calheiros CML, Campelo-Junior EB, Duarte EM, Heukelbach J, Feldmeier H (2010). Occurrence of the off-host life stages of Tunga penetrans (Siphonaptera) in various environments in Brazil. Ann Trop Med Parasitol.

[11] Heukelbach J, Feldmeier H, Wilcke T, Eisele M (2002). Ectopic localization of tungiasis. Am J Trop Med Hyg.

[12] Muehlen M, Heukelbach J, Wilcke T, Winter B, Mehlhorn H (2003). Investigations on the biology, epidemiology, pathology and control of Tunga penetrans in Brazil: II. Prevalence, parasite load and topographic distribution of lesions in the population of a traditional fishing village. Parasitol Res.

[13] Feldmeier H, Heukelbach J, Ugbomoiko US, Sentongo E, Mbabazi P, von Samson-Himmelstjerna G (2014). Tungiasis—a neglected disease with many challenges for global public health. PLoS Negl Trop Dis.

[14] Feldmeier H, Heukelbach J. Tungiasis. Orphanet Encycl. 2004;1–4. [Internet] Available at: http://www.orpha.net/data/patho/GB/uk-Tungiasis.pdf.

[15] Elson Lynne, Wright Katherine, Swift Jennifer, Feldmeier Herman (2017). Control of Tungiasis in Absence of a Roadmap: Grassroots and Global Approaches. Tropical Medicine and Infectious Disease.

[16] Tsebeni S (2016). Prevalence and risk factors associated with tungiasis in Mayuge district, Eastern Uganda. Pan Afr Med J.

[17] Muehlen M, Feldmeier H, Wilcke T, Winter B, Heukelbach J (2006). Identifying risk factors for tungiasis and heavy infestation in a resource-poor community in Northeast Brazil. Trans R Soc Trop Med Hyg.

[18] Ugbomoiko US, Ariza L, Ofoezie IE (2007). Risk Factors for Tungiasis in Nigeria: Identification of Targets for Effective Intervention. PLoS Negl Trop Dis.

[19] Heukelbach J, Wilcke T, Harms G, Feldmeier H. Seasonal variation of Tungiasis in an endemic community. Am J Trop Med Hyg [Internet] 2005;72(2):145–9. Available at: http://citeseerx.ist.psu.edu/viewdoc/download?doi=10.1.1.532.7721&rep=rep1&type=pdf 15741550

[20] Dassoni Federica, Polloni Ilaria, Margwe Sulle Baltazar, Veraldi Stefano (2014). Tungiasis in Northern Tanzania: a clinical report from Qameyu village, Babati District, Manyara Region. The Journal of Infection in Developing Countries.

[21] Kamau T, Ngechu R, Haile ZTMJ (2014). An exploration of factors associated with jigger infestation (Tungiasis) among residents of Muranga North District. Kenya Int J Heal Sci Res.

[22] Heukelbach È, Oliveira ÂS, De HG, Feldmeier H (2001). Tungiasis: a neglected health problem of poor communities. Trop Med Int Heal.

[23] Bourée P, Ossé L, Rabenandrasana F (2009). Tungiasis, an uncommon ectoparasitic disease. Rev Prat.

[24] Ariza L, Seidenschwang M, Buckendahl J, Gomide M, Feldmeier H (2007). Tungiasis: a neglected disease causing severe morbidity in a shantytown in Fortaleza, state of Ceara´. Rev Soc Bras Med Trop.

[25] Feldmeier H, Sentongo E, Krantz I (2012). Tungiasis (sand flea disease): a parasitic disease with intriguing challenges for public health. Eur J Clin Microbiol Infect Dis.

[26] Feldmeier H, Eisele M, Sabóia-moura RC, Heukelbach J (2003). Severe Tungiasis in underprivileged communities: case series from Brazil. Emerg Infect Dis.

[27] Ariza L, Wilcke T, Jackson A, Gomide M, Ugbomoiko US, Feldmeier H (2010). A simple method for rapid community assessment of tungiasis. Trop Med Int Heal.

[28] Mwangi JN, Ozwara HS, Gicheru MM. Epidemiology of *Tunga penetrans* infestation in selected areas in Kiharu constituency, Murang’a County, Kenya. Trop Dis Travel Med Vaccines [Internet]. 2015;1(1). Available at: 10.1186/s40794-015-0015-4 10.1186/s40794-015-0015-4 PMC5530937 28883944

[29] Ngunjiri et al. Impact of Tungiasis on acquisition of basic education among children aged 5–14 years in Murang ’ a County, Kenya. Int J Sci Res Innov Technol. 2015;2(6):137–8.

[30] Bucyensenge JP. Southern Province leaders launch hygiene campaign. Rwanda: Southern Province; 2015. Available at: https://www.newtimes.co.rw/section/read/184907. Accessed 26 July 2019.

[31] Datar, G. Earthenable, Kigali, Rwanda. Personal communication, 2017. Available at: https://www.google.com/search?client=firefoxd&q=Datar%2C+G.+Earthenable%2C+Kigali%2C+Rwanda.+Personal+communication%2C+2017. Accessed 28 Jul 2019.

[32] National Institute of Statistics of Rwanda (NISR) M of F and EP (MINECOFIN) [Rwanda]. Fourth Population and Housing Census, Rwanda. Fourth Popul Hous Census, Rwanda, 2012. 2012;(May):1–18.

[33] Al-Subaihi AA (2003). Sample size determination. Influencing factors and calculation strategies for survey research. Saudi Med J.

[34] Hernandez DJ, Napierala JS. Mother’s education and children’s outcomes: how dual-generation programs offer increased opportunities for America’s families. Found Child Dev. 2014;4–7. [Internet] Available at: https://files.eric.ed.gov/fulltext/ED558149.pdf.

[35] Maïga EWH. The Impact of Mother’s Education on Child Health and Nutrition in Developing Countries: Evidence from a Natural Experiment in Burkina Faso. African Cent Econ Transform. 2015;(January):1–53.

[36] Bilate Angelina B., Lafaille Juan J. (2011). It Takes Two to Tango. Immunity.

[37] Mazigo HD, Bahemana E, Dyegura O, Mnyone LL, Kweka EJ, Zinga M (2018). Jigger flea infestation ( tungiasis ) in rural western Tanzania : high prevalence and severe morbidity severe tungiasis in Western Tanzania : case series. J Public Health Africa.

[38] Ugbomoiko US, Ariza L, Babamale AO, Heukelbach J (2017). Prevalence and clinical aspects of tungiasis in south-west Nigerian schoolchildren. Trop Dr.

[39] Wiese Susanne, Elson Lynne, Reichert Felix, Mambo Barbara, Feldmeier Hermann (2017). Prevalence, intensity and risk factors of tungiasis in Kilifi County, Kenya: I. Results from a community-based study. PLOS Neglected Tropical Diseases.

[40] Mwangi JN, Ozwara HS, Gicheru MM. Epidemiology of *Tunga penetrans* infestation in selected areas in Kiharu constituency, Murang’ a County , Kenya. Trop Dis Travel Med Vaccines [Internet]. 2015;1–6. Available at: http://dx.doi.org/10.1186/s40794-015-0015-4 10.1186/s40794-015-0015-4 PMC5530937 28883944

[41] Ngunjiri J, Keiyoro PN (2015). Quantifying burden of disease caused by Tungiasis using disability adjusted life years metric among the children aged 5–14 years in Murang ′ a County , Kenya. Int Res J Public Environ Heal.

[42] Feldmeier H, Wilcke T (2002). Regina Sansigolo Kerr-pontes L, César Sabóia Moura R, Heukelbach J. high prevalence of tungiasis in a poor neighbourhood in Fortaleza, Northeast Brazil. Acta Trop.

[43] Wanzala M, Silali MB (2016). Factors influencing prevention of Tungiasis infestation in Tshiatsala division of Butere Sub County – Kenya. SMU Med J.

[44] Ugbomoiko Uade Samuel, Ariza Liana, Ofoezie Ifeanyi Emmanuel, Heukelbach Jörg (2007). Risk Factors for Tungiasis in Nigeria: Identification of Targets for Effective Intervention. PLoS Neglected Tropical Diseases.

[45] WHO. Neglected tropical diseases Prevention, control, elimination and eradication Report by the Secretariat. Doc WHO/CDS/NTD/20061 [Internet]. 2013;2012(March 2013):1–40. Available at: http://www.who.int/neglected_diseases/A66_20_Eng.pdf

[46] Girma M, Astatkie A, Asnake S (2018). Prevalence and risk factors of tungiasis among children of Wensho district, southern Ethiopia. BMC Infect Dis.

[47] Mutebi F, Krücken J, Feldmeier H, Waiswa C, Mencke N. Tungiasis-associated morbidity in pigs and dogs in endemic villages of Uganda. Parasit Vectors [Internet]. 2016;1–9. Available at: http://dx.doi.org/10.1186/s13071-016-1320-0 10.1186/s13071-016-1320-0 PMC4729147 26817587

[48] Feldmeier H, von Samson-Himmelstjerna G, Waiswa C, Bukeka Muhindo J, Krücken J, Mutebi F (2016). Successful treatment of severe Tungiasis in pigs using a topical aerosol containing Chlorfenvinphos, Dichlorphos and gentian violet. PLoS Negl Trop Dis.

[49] Waruguru C, Mwaniki P, Karama M, Muthami L (2015). Prevalence of Tungiasis and its associated factors among residents of Kipkelion west Sub-County; Kericho County, Kenya. Int J Heal Sci Res.

[50] Beatrice Makena TM (2014). Jigger infestation a menace to children ’ s school attendance. J Educ Pract.

