# Supplementary material for: Factors associated with tungiasis among primary school children: a cross-sectional study in a rural district in Rwanda
Source: BMC Public Health. 2019 Aug 29;19:1192. doi: 10.1186/s12889-019-7481-y (PMC6716852; doi:10.1186/s12889-019-7481-y)
Supplement: Supplementary file 1 — Research questionnaire (DOCX 49 kb) [file 12889_2019_7481_MOESM1_ESM.docx]

# Additional file1 Research questionnaire

Study title: **Factors associated with tungiasis among primary school children: a cross-**

**sectional study in a rural district in Rwanda.**

| 1.Sex of the child | Male  Female |
| --- | --- |
| 2.Age of the child (years) | _____________ |
| 3.Respondent | Mother  Father |
| 4.Maternal education | No schooling  Primary  Secondary  University  Other (specify)____________________ |
| 5.Father occupation | Farmer  Monthly salaried employee  Trader  Occasional occupation  Other (specify)____________________ |
| 6.Tungiasis status in a child | Infected  Non-infected |
| 7.Clinical manifestations suggesting tungiasis infestation | - Skin lesions (nodules with black centres, suppurative ulcers or punctiform cavities, itching spots, walking difficulty, oedema and skin redness around lesions, loss of toenails or deformed nails, other to be specified):  ___________________________________________________________  ___________________________________________________________  ___________________________________________________________  - Location of skin lesions (feet, toes, fingers, interdigital spaces, other to be specified): ____________________________________________________________  ____________________________________________________________  ________________________________________________________  - History of clinical manifestations (duration in days or weeks or months, chronology of manifestations, other people having the same manifestations at home, similar manifestations in the past, etc.):  ____________________________________________________________  ____________________________________________________________  ____________________________________________________________  ____________________________________________________________  ____________________________________________________________  - Therapeutic measures taken (removal of tunga penetrans using a needle or thorn, use of desinfectant on lesions, seeking care at a health facility, other forms of treatment used to be specified). ____________________________________________________________  ____________________________________________________________  ____________________________________________________________  - Evolution of lesions (improved/healed lesions, sequellae such as nails deformity, etc.):  _________________________________________  _____________________________________________________________  _____________________________________________________________ |
| 8. Body hygiene | Clean feet  Dirty feet (unwashed, covered by dust) |
| 9. Clothes hygiene | Clean clothes  Dirty clothes (unwashed, covered by dust) |
| 10. Wearing shoes | Always wear shoes (at home or elsewhere)  Irregular wearing of shoes (only when going to school, to the church, or other social events)  Never wear shoes |
| 11. Possession of domestic animals at home | Yes  No  If yes, specify the type and number of animals (goat, cow, pig, hens, rabbit, cats, dog, etc.):  _____________________________________________ |
| 12. Sharing house with domestic animals | Yes  No  If yes, specify which type of domestic animals:  ____________________________________ |
| 13. Plastering of the house floor | Earthen floor  Yes  No  Cemented floor  Yes  No  Other  Please, specifyother material covering the floor if any:  ____________________________________________________________ |
| 14. School attendance | Regular attendance (never or rarely missed in the class)  Irregular attendance (missed in the class one or more days per week repeatedly):  Yes  No  If irregular attendance, specify the number of days missed in the class per week (within a period of last six months):  ____________________________________________________________ |
| 15. Reasons for irregular school attendance | Difficulty of walking due to pain  Yes  No  Itching disturbing the concentration in class  Yes  No  Isolation or stigma from classmates  Yes  No  Other reason  Please, specify other reason if any: ____________________________________________________________  ____________________________________________________________  ____________________________________________________________  ____________________________________________________________  Not applicable (regular attendance) |
| 16. School performance | Average score obtained from the first two trimesters of the current school year (in percentage):  First trimester: _____________________________________________________________  Second trimester: _____________________________________________________________  Average of the first two trimesters:  _____________________________________________________________ |
